# Topical Application of Manuka Honey for the Treatment of Non-Healing Venous Leg Ulcers

**DOI:** 10.3390/ph18020149

**Published:** 2025-01-23

**Authors:** Marek Kucharzewski, Kinga Spyrka, Ewa Rojczyk, Jakub Brela

**Affiliations:** 1 Wladyslaw Bieganski Collegium Medicum, Jan Dlugosz University in Czestochowa, 42-200 Czestochowa, Poland; kucharzewskimarek@poczta.onet.pl (M.K.); ikingaxd@gmail.com (K.S.); j.brela@ujd.edu.pl (J.B.); 2 Surgical Outpatient Clinic, John Paul II District Hospital in Włoszczowa, 29-100 Włoszczowa, Poland; 3 Faculty of Medicine, Academy of Silesia in Katowice, 40-555 Katowice, Poland

**Keywords:** Manuka honey, venous leg ulcer, wound healing, microorganisms

## Abstract

**Background/Objectives**: Issues related to the chronic venous leg ulcer (VLU) treatment and prevention of recurrences remain the subject of research, but so too do common clinical problems in daily medical practice. Due to its medicinal properties, Manuka honey is increasingly used in the treatment of wounds of various origins. The aim of the study was to investigate the effectiveness of Manuka honey for the topical treatment of non-healing, chronic, venous leg ulcers. **Methods**: Eighty patients with chronic VLU participated in the study and were randomized into two equinumerous groups. In group 1, patients were treated with topical Manuka honey application and short stretch bandage compression, whereas, in group 2, antimicrobial calcium alginate wound dressing + Ag was used instead of Manuka honey. The efficacy of both treatment methods was compared. **Results**: The ulcerations in patients from group 1 have healed completely after up to seven weeks of therapy in all cases. In contrast, in all patients from group 2, the healing process was longer but completed successfully after up to 14 weeks of the therapy. The process of wound cleaning from microorganisms was also faster in group 1, as well as the reduction in ulcer area during treatment. **Conclusions**: It was found that the topical administration of Manuka honey may be a promising alternative to traditional methods of non-healing VLU treatment.

## 1. Introduction

Venous leg ulcer (VLU) has become a common medical problem among elderly patients. Non-healing VLU is defined as a chronic wound of the lower limb that does not heal after 3 months of therapy or is still not fully healed after 12 months of treatment. Chronic leg ulcers of various origin (70% of which are VLUs) cause long-term illness in older adults and the condition places a significant burden on health service resources. It affects 0.6–3% of those aged over 60 years, increasing to over 5% of those aged over 80 years, whereas the overall prevalence of chronic leg ulcers in the community ranges from 1.9% to 13.1%. Moreover, the patients suffer from multiple comorbidities and the illness has a remarkable impact on life quality [1,2,3].

It is estimated that the annual incidence of leg ulcers is 3.5 per 1000 individuals in the UK and 0.2 per 1000 in Switzerland. In the USA, the prevalence of VLU is between 500,000 and 600,000 and increases with age [1]. Jawień et al. reported that active or healed leg ulcers are found in 1.53% of patients visiting primary care institutions in Poland [4].

As found in previous studies and practice, compression therapy and local therapy are helpful ulcer treatment methods. However, the rate of wound healing complications after their application is still very high. As such, TIME strategy (Tissue debridement, Infection/Inflammation, Moisture balance, Edge effect) and proper wound dressing are of great importance and various hydrogels and hydrocolloids, as well as alginates and foams help in proper treatment [5].

Honey has been used in wound care since ancient times. It was found in scriptures from Sumer (from about 2000 BC) and in Smith papyrus (1700 BC) as a prescription for treating wounds. According to the Ebers papyrus (1550 BC), it was included in 147 prescriptions for external applications. Similarly, Chinese and Indian ayurvedic medicine used honey for many purposes, not only for wound healing but also against different internal and external infections. The ancient Greeks and Romans, represented by thinkers like Homer, Hippocrates, Dioscorides, and Pliny, gave honey much attention, emphasizing that it is a product that should be regularly used to preserve health and vigor [6]. Honey is now being rediscovered by modern medicine as a topical agent for wound treatment, especially where conventional modern therapeutic agents fail. It is one of the most complex and valuable natural biological products that has been widely used for its therapeutic effects.

Honey is a mixture of sugars prepared by honey bees from the natural sugar solutions—called nectar. The bee performs an inversion of the sucrose in the nectar, which increases the attainable density of the final product. Then, thanks to the addition of enzymes and the evaporation of water, honey bees transform it into a sweet liquid. There are two main types of honey: apiary honey and forest honey. Apiary honeys are produced in apiaries by Indian *Apis cerana indica* bees and the European *Apis millifera* bees, they are transparent and free from foreign materials. In contrast, forest honeys are produced by the rock bee, *Apis dorsata*, or from wild nests of *Apis cerana indica* in forests, and they are turbid owing to the abundant presence of pollen, wax, brood (bee larvae), and plant materials [7].

Natural honey is mainly made up of sugar and water, with sugar accounting for over 95% of the dry matter. Generally, it is a complex of carbohydrates; however, over 200 health-promoting substances have been found in natural honey, including various vitamins, flavonoids, minerals, and enzymes, as well as amino acids, organic acids, and volatile chemicals. It is important to note that numerous factors affect the quality of honey, including plant type, location, and season, as well as processing method and microbiological characteristics. Thus, such factors also influence the medicinal properties of honey [8,9,10].

Honey has numerous health-promoting properties (like antioxidant and anti-inflammatory properties), it contains a wide range of vitamins, amino acids, and minerals, glucose oxidase producing hydrogen peroxide (H_2_O_2_) in diluted honey, and also gluconic acid (inhibited in undiluted honey) resulting in the pH level of honey ranging from 3.2 to 4.5. Moreover, honey is known to have strong antibacterial properties because of high osmolarity—it was shown, that in undiluted honey, bacterial growth is prevented. Furthermore, honey is non-toxic, non-allergenic, does not stick to wounds, and helps rapid wound healing because of its moisture content. Most importantly, honey is cheap and, therefore, the application of honey as a topical treatment is very beneficial in regard to infection control and wound management [5,11,12,13,14,15,16,17].

Manuka honey (the Maori name for the New Zealand/Australian tea tree/bush *Lepto-spermum scoparium*) is a monofloral honey derived from the tea tree by honey bees *Apis melifera* [18]. It has antibacterial properties and, as stated by Moore, it has “very special healing properties”, which makes it “the best natural antibiotic in the world”. Medical-grade Manuka honey is prepared very purely and undergoes very specific and rigorous quality control [6,19,20].

Due to its properties, Manuka honey has many applications, including the treatment of burns as well as the treatment of external ulcers. It was shown that not only does honey produce a clean, granulating wound bed, but it also has a positive impact on wound repair because of its nutritional activity. Such activity includes supporting the osmotic flow of lymph towards the wound, the intake of carbohydrates (which are easily metabolized) as well as amino acids, vitamins, and minerals, all of which support wound healing. The high osmotic property of honey results in interstitial fluid drainage, promoting tissue regeneration, which (observed in areas of angiogenesis) is seen as granulation [21,22,23,24,25]. Honey may also have an influence on whether surgical debridement or adherence of dressing to the wound is necessary, as osmotic flow impacts the removal of waste and debris from the wound. As honey forms a fluid layer on wound surfaces after application, wounds may be rinsed, resulting in painless dressing changes, which do not involve reparative tissue damage [13,14,16,17,20,26].

After applying Manuka honey to wounds, healing time decreases, wound surface decreases, and tissue repair is accelerated [13,16,27]. What is more, Manuka honey dressings can be applied directly to dry and also to moist wounds. They are frequently used instead of modern antibacterial wound dressings containing ionic silver, especially in critically colonized wounds, i.e., those with a bacterial load that exceeds the host’s defense capabilities [18,26,27]. An example of such a wound is a diabetic foot ulcer (DFU). It was shown that honey is a cost-effective and safe option for DFU management that reduces the time of healing, as well as amputation or hospitalization rates [21,28,29,30]. However, another type of difficult-to-heal wound—VLU—is not so widely described in the literature in the context of using honey for its treatment. There are only some promising case series describing less than ten patients with VLUs, who were successfully treated with Manuka honey within several weeks [31,32]. On the other hand, a systematic review of the literature concerning medical-grade honey used in patients with VLUs gives contradictory results [33].

Thus, in order to complete data on this topic, the aim of the study was to determine the effectiveness of Manuka honey in a topical dressing for the treatment of non-healing VLUs, as well as to assess the impact of such dressing on the wound bacterial microbiota. Despite extensive knowledge about the healing effects of honey, our study is the first one that concerns only one, specific type of wound—VLUs. This study is a kind of continuation of an earlier experiment on the effect of propolis on VLU healing [34].

## 2. Results

In the course of the study, none of the patients rejected the proposed treatment in both groups.

### 2.1. Complete Healing Time

In all 40 patients in group 1, the ulcers healed successfully (i.e., completely) within the first 7 weeks of treatment by topical Manuka honey application. Ulcer healing was also observed in all the cases in group 2 (antimicrobial calcium alginate wound dressing + Ag). However, within the first 7 weeks of treatment, complete healing was found only in 12 out of 40 patients in this group and the time up to the complete healing of the last two cases was significantly longer—14 weeks. (Table 1). The maximum time of the ulcer healing in group 1 was significantly shorter (49 days) than in group 2 (98 days); *p* < 0.05.

### 2.2. Ulceration Area and a Rate of Wound Area Reduction

**Figure 1:**
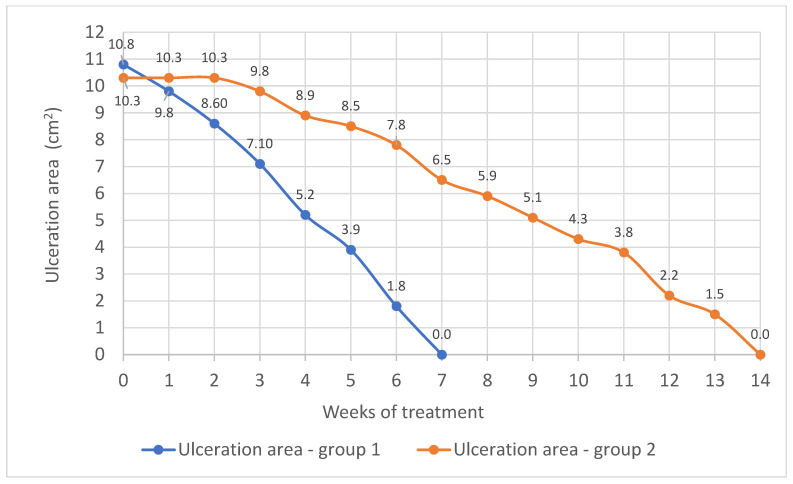
Changes in ulceration area in subsequent weeks of treatment. Group 1—Manuka honey; group 2—standard treatment.

**Figure 2:**
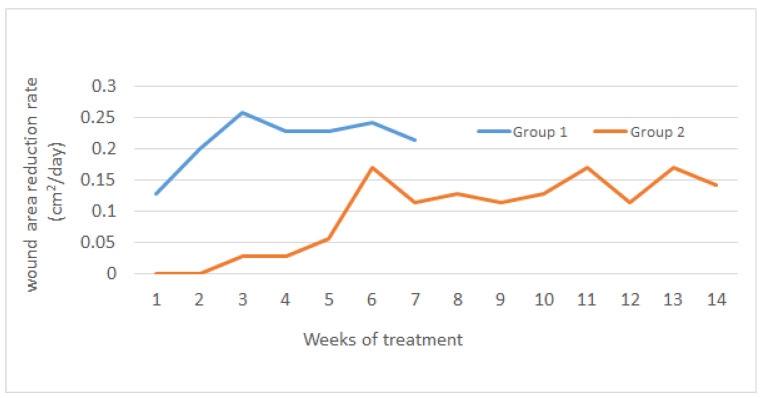
The rate of wound area reduction depending on the treatment time. Group 1—Manuka honey; group 2—standard treatment.

The mean ulceration area in groups 1 and 2 in the course of the treatment is presented in Figure 1, whereas Figure 2 presents changes in the rate of wound area reduction over time.

In group 1, the mean ulceration area at the beginning of the study was 10.8 cm^2^. After 7 days of treatment, the average ulceration area diminished by 1.0 cm^2^ and the rate of reduction of the wound area (v_1_) was 0.142 cm^2^/day. After another 7 days, the rate with which the ulcer area was decreasing equaled 0.171 cm^2^/day, and the mean area of the wound decreased by 1.2 cm^2^. By this time, the ulcers in four patients have been healed. After the following 14 days of using the Manuka honey dressing, the ulcers of a further 12 women and 6 men healed completely, and the mean wound area of the remaining patients decreased by 3.4 cm^2^. The rate at which the wound area decreased was 0.214 cm^2^/day (first week) and 0.271 cm^2^/day (second week). After a further 7 days, the ulcers of 7 more patients healed, the rate of wound area reduction was 0.186 cm^2^/day and the mean area of the wound decreased by 1.3 cm^2^. In the next 7 days of treatment, the mean ulceration area diminished by 2.1 cm^2^, and the rate of wound area reduction was 0.3 cm^2^/day. The ulcers of four women and three men healed completely. The healing of the ulcers in the last patients was completed after the next 7 days of treatment. The rate of decrease in the wound area in that period was 0.257 cm^2^/day.

Group 2 consisted of 40 patients with a mean ulceration area of 10.3 cm^2^. Over the following 2 weeks of treatment, the surface area of the ulcers did not change and was 10.3 cm^2^. After a further 7 days of treatment, the mean wound area decreased by only 0.5 cm^2^ and the rate at which it decreased (v_2_) was 0.071 cm^2^/day. After a further 7 days, the rate of ulceration area reduction was also 0.129 cm^2^/day and the mean wound area was 8.9 cm^2^. The ulcers of four women and two men healed completely. After the following 14 days of treatment, the mean ulcer area decreased by 1.1 cm^2^ and the rate with which the wound surface decreased was 0.057 cm^2^/day (first week) and 0.1 cm^2^/day (second week). The mean ulcer area of the other patients decreased (7.8 cm^2^). After a further 7 days, the rate of the ulceration surface reduction was 0.186 cm^2^/day and the mean wound area was 6.5 cm^2^. The ulcers in seven patients were healed. After a further 7 days of treatment, the mean wound area decreased by 0.6 cm^2^ and the rate at which it decreased was 0.086 cm^2^/day. After a further 14 days, the mean wound area was 1.6 cm^2^ and the reduction in the wound area was 0.114 cm^2^/day for both weeks. The ulcers of eight women and four men healed completely. After the following 14 days of treatment, the mean ulcer area was 2.1 cm^2^, and the rate of wound area reduction was 0.071 cm^2^/day (first week) and 0.229 cm^2^/day (second week). The ulcers of seven patients healed completely. The ulcerations of the remaining five patients in this group healed after a further 14 days of treatment and the rate at which the ulceration area decreased was 0.1 cm^2^/day (first week) and 0.214 cm^2^/day (second week).

Thus, a significantly faster rate of wound area reduction was observed in group 1 (treated with Manuka honey). In group 2, the healing process accelerated only from the sixth week of treatment. However, even after such a long time, the rate of wound area reduction was comparable to the value for group 1 in the first week of treatment (Figure 2).

### 2.3. Microbiota Analysis

The qualitative analysis of microbiota isolated from the surface of venous leg ulcers is shown below. Diagnostic cultures performed before treatment indicated that all wounds in both patient groups were infected. After three weeks of wound therapy, bacterial cultures were detected in 18 patients from group 1 and in 35 patients from group 2. After five weeks of treatment, the cultures made from ulcer swabs were positive for bacteria in six patients of group 2. All ulcerations in patients of group 1 treated with Manuka honey dressing did not support culturable microorganisms. Generally, 12 species of bacteria were grown from all samples taken before treatment, but only seven of the most common ones are presented in Table 2. In ulcers of the patients treated with calcium alginate wound dressing + Ag the incidence of individual isolates did not differ substantially from the results obtained in patients in group 1 (treated with Manuka honey). In both groups, the most common isolated microorganisms were *Staphylococcus aureus, Echerichia coli, Pseudomonas aeruginosa*, and *Proteus mirabilis*, whereas *Streptococcus β-haemoliticus, Enterococcus faecalis*, and *Klebsiella pneumoniae* were rarely isolated species. Before treatment, 182 strains of different bacteria were isolated from the wounds of patients in group 1, whereas, in group 2, there were 194. The application of Manuka honey dressing for three weeks resulted in reducing the number of bacterial strains to 40, and, after 5 weeks, no bacterial strain was isolated. In swabs from the wounds of patients treated with topical antimicrobial calcium alginate wound dressing, 131 strains were cultured after 3 weeks, and 78 after 5 weeks (Table 2).

## 3. Discussion

Despite the vast improvement in epidemiological and pathophysiological knowledge, one of the largest clinical concerns in phlebology includes VLUs. The number of patients suffering from this complication remains high; therefore, there is a need to search for more effective treatment strategies [34].

Both systemic and local factors affect the wound-healing process. That is why it is highly important that topical medications have a positive effect on healing. One of the promising natural products supporting wound healing is Manuka honey. In recent years, the molecular mechanisms of its activity in the context of healing promotion have become increasingly better understood. Leukocyte stimulation greatly affects the healing rate by the release of cytokines and growth factors, stimulating keratinocyte transcription of TNF-α, IL-1β and TGF-β genes resulting in tissue repair activation [35,36]. Apart from interactions with the immune system, honey induces Ca^2+^ entry into keratinocytes as a result of H_2_O_2_ release in a reaction mediated by glucose oxidase. Consequently, wound closure is triggered [22,23]. Another interesting mechanism of anti-inflammatory honey activity is the inhibition of the nuclear factor kappa B (NF-кB) pathway, which decreases inflammatory mediators, such as cyclooxygenase-2 (COX-2) and cyclooxygenase-1 (COX-1) [21,24].

**Figure 3:**
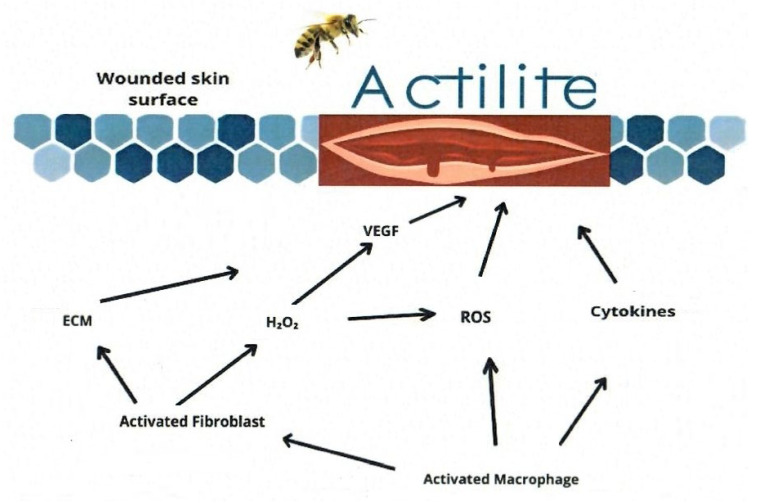
Most important molecular changes in wound area after honey dressing Actilite application [21,22,24].

The most important molecular factors modulated by honey in the context of wound healing promotion are summarized in Figure 3.

All these physical and chemical factors give honey unique properties as a wound dressing. Indeed, it is probably due to the abovementioned properties that, in our study, faster healing of VLUs was observed in patients treated with Manuka honey than in patients from the group receiving calcium alginate wound dressing + Ag. Honey contributed to wound healing within a maximum of 7 weeks as compared to a maximum of 14 weeks in patients treated with calcium alginate wound dressing + Ag.

Other research groups observed similar results. For example, the healing outcomes of relatively small, short-duration venous ulcers, treated with multi-layer compression and calcium alginate dressings impregnated with Manuka honey (n = 187) or primary dressings according to “usual care” changed as needed (n = 181) were compared by Jull et al. Within a 12-week period, 55.6% of patients belonging to the Manuka honey group and 49.7% of “usual care” patients (*p* = 0.258) experienced fully healed wounds [35]. A hydrocellular foam dressing impregnated with either Manuka honey (n = 54) or an amorphous hydrogel (n = 54) on venous ulcers of longer duration, covered with at least 50% slough, was used by Gethin and Cowman. It was observed that those patients had larger venous ulcers than the patients studied by Jull et al., despite a weekly application of dressings with up to four layers of compression bandaging for a time span of 4 weeks [37]. After using Margolis’ [38] standardized adjustment for venous ulcer areas > 5 cm^2^ and a duration > 6 months, a greater percentage of patients in the Manuka honey group (*p* = 0.025) recovered at the 12-week period (*p* = 0.037) compared to the hydrogel control group, and earlier epithelialization (*p* = 0.042) was observed [37]. Similarly, the case series of nine patients with wounds of various aetiologies confirmed that medical-grade honey can be an effective alternative for antibiotics in locally infected wound treatment [39].

Conflicting results were presented by Ingle et al. in a prospective, randomized, double-blind study. In this study, the effects of honey and IntraSite gel were compared. It was observed that even after adjusting for wound size, the healing time of shallow wounds treated with honey did not significantly differ from the healing time of shallow wounds treated with IntraSite gel. In the case of shallow wounds, those treated with honey healed 2.8 days sooner, while, in regard to abrasions, there was no significant difference. It was concluded that there was no significant difference between IntraSite gel and honey when used as treatment agents [40].

Du Toit and Page used an in vitro tissue explant culture model to examine how honey and silver-impregnated dressings influence the morphology of two key cellular components of the wound bed, i.e., keratinocytes and fibroblasts. It was shown that the honey-impregnated dressings promoted new tissue regeneration [41]. Another study, which compared silver-sulphadiazine with the honey gel provided similar results—the honey gel significantly stimulated re-epithelialization, whereas silver sulphadiazine significantly reduced it [42].

Wounds are exposed to a number of microbes found on the skin and in the external environment. If the repair process does not run properly and wounds do not heal, they may become infected, which leads to the formation of biofilm in the wound. The biofilm contains many species of bacteria, which strongly adhere to the wound bed and to each other [43]. Topical application of the dressing with Manuka honey maintains a moist environment in the wound and shows antibacterial activity against a broad range of pathogens, such as aerobic and anaerobic bacteria, fungi, and viruses [44,45,46]. Another advantage (over antibiotics) is also the long-lasting antibacterial effect and a significantly lower tendency to induce bacterial resistance.

Numerous studies [45,46,47,48,49,50,51] have shown that Manuka honey inhibits more than 60 species of bacteria, including anaerobic Gram-positive bacteria and Gram-negative bacteria, and even a few yeast species of the *Aspergillus* and *Penicillium* genus. The antibacterial properties of honey are due to its high osmolarity and the production of H_2_O_2_. Its secretion from the dressing is slow and continuous, which results in the elimination of microorganisms; however, it is not cytotoxic to the tissues surrounding ulceration. The acidic environment in the wound produced by honey also inhibits the growth of pathogens in the skin loss. The low pH increases the release of oxygen from hemoglobin in the wound, which stimulates angiogenesis and cytokine secretion and consequently increases the rate of granulation [21,22,23,24,25]. Cooper et al. studied the antibacterial effectiveness of three honeys (including artificial honeys) on 17 strains of *Pseudomonas aeruginosa* isolated from the wounds of patients. Manuka honey showed antibacterial properties at a concentration of 6.8–7.5%, and two other (artificial) honeys required a concentration of 17% and 22%, respectively, to inhibit the development of all 17 strains [52]. Similar results were obtained by many other authors [27,53,54,55], concerning many types of chronic wounds, including VLUs.

Our results confirm the high effectiveness of Manuka honey in combating bacterial colonization of the wound: after 5 weeks of treatment, there were no culturable bacterial strains isolated in the group receiving honey dressings (as opposed to the traditionally treated group). It is worth mentioning that Manuka honey successfully eradicated *Enterococcus faecalis*, which is a non-pathogenic commensal when isolated from the healthy skin of a healthy subject. However, in patients with VLUs, it is highly pathogenic and seriously delays wound healing. It can be a source of local wound infection leading to sepsis.

To sum up, there are many studies comparing various applications of Manuka honey with other methods for the topical treatment of burns and DFUa. However, few studies examine the treatment of VLUs with honey. Thus, in one of our experimental groups, we used the Manuka honey dressing to determine how it affects the treatment of VLUs. Despite the encouraging effects achieved using Manuka honey therapy of non-healing VLUs, further, more detailed studies concerning honey implementation should be performed, including randomized controlled trials, comparisons with other methods of treatment, and involving larger experimental groups.

## 4. Materials and Methods

### 4.1. Patients

The study was conducted at the Surgical Outpatient Clinic of the John Paul II District Hospital in Włoszczowa in the period from February 2022 to September 2024.

The experiment comprised 80 patients with non-healing venous leg ulcer (VLU) and a history of active crural ulcer ≥12 months as well as a lack of complete healing despite previous non-surgical treatment. Each patient provided information about ulcer treatment and other significant medical problems. All patients were previously treated by a general practitioner with elastic bandage compression stocking along with wound antiseptic lavage and local application of traditional dressing, like hydrogel and hydrocolloid. However, none of these methods led to the complete healing of the wound within the pre-randomization period.

After clinical examination, the venous origin of the ulcer was confirmed with the help of the venous duplex Doppler sonography and ankle-brachial index (ABI) measurement (in all the cases the ABI was ≥0.8). Patients with previous or active deep vein thrombosis were excluded from the study. Additional exclusion criteria were diabetes, chronic or critical leg ischemia, contraindications to compression therapy, orthosis immobilization or plastic cast, paresis related to stroke or paraplegia, chronic cardiac failure with peripheral swelling, systemic infection, clinically significant peripheral atherosclerotic occlusive disease, and previous honey implementation. Moreover, patients treated pharmacologically with sulodexide or other anticoagulants as well as patients on pentoxifylline or prostaglandins were not included in the study.

The patients qualified for the study were divided into two groups using a randomized method. Similarly, randomized numbers were given to patients according to the order of inclusion into the study (group 1 included the patients with odd numbers, and group 2 those with even numbers).

Group 1 included 27 women and 13 men aged 49–79 years (mean 56.5 years). Their ulceration area varied from 6.8 cm^2^ to 17.4 cm^2^ (mean 10.8 cm^2^). The ulcer location of 14 patients was on the left crus, and, for 26 patients, it was on the right one. The wound persisted at the start of therapy for 17–29 months (mean 21 months). Thirty patients were fully mobile, and ten women had limited mobility. Body mass index (BMI) ranged from 23.6 to 39.7 kg/m^2^ (mean 31.2 kg/m^2^). It was >30 kg/m^2^ in 10 women and 6 men (Table 3).

Group 2 included 28 women and 12 men aged from 51 to 75 years (mean 58.1 years). The ulceration area varied from 6.6 to 17.8 cm^2^ (mean 10.3 cm^2^), the duration of wound occurrence from 18 months to 31 months (mean 22 months). In 16 patients, the ulcers were located on the left crus, and, in 24 patients, they were on the right one. Full mobility was possible in 29 patients and limited mobility in 11 patients. BMI ranged from 24.8 to 40.2 kg/m^2^ (mean 29.8 kg/m^2^). It was >30 kg/m^2^ in 9 women and 8 men (Table 3).

Importantly, there were no statistically significant differences between groups in the parameters presented in Table 3.

Each ulcer was classified by wound morphology, severity, and location. A systematic description of wound and limb appearance was recorded, including edema, erythema, exudation, granulation, and the presence of fibrin or eschar. Moreover, based on clinical and sonographic results, the chronic venous disease of each patient was classified according to CEAP classification (Table 4).

### 4.2. Procedures

All patients underwent ambulatory treatment and were examined by a physician every week until the complete healing of the ulceration. The drug therapy in both groups followed a standard regimen. All patients received micronized flavonoid fraction (450 mg diosmin, 50 mg hesperidin), and 2 tablets of 500 mg once a day.

The ulcers were thoroughly debrided together with the removal of fibrin, eschar, and, if necessary, necrotic tissue. The necrotic tissues were removed from ulcerations through surgery or with the help of enzyme-containing ointment and antiseptic lavage.

For the local treatment in group 1, a topical application of Manuka honey dressing (Activon Honey, Advancis Medical, Nottingham, UK) was used. The ulcers were rinsed with physiological sodium chloride solution and then the Manuka honey dressing was applied. Gauze pads were then placed on the ulcer and the leg was bandaged with a non-elastic supportive bandage. The compression short-stretch bandages were applied to all patients in this group. Specifically, it was a spiral two-layer bandaging technique with a 10 cm wide bandage applied from toe to knee. Manuka honey dressing was changed every day until the ulcer was healed. For the proper measurement of compression pressure, the Kikuhime device pressure sensor was used and a pressure of 25–35 mm Hg was applied.

The patients in group 2 were treated with antimicrobial calcium alginate wound dressing + Ag (Suprasorb^®^ A + Ag, Lohmann and Rausche, Pabianice, Poland). After rinsing the wound with physiological sodium chloride solution, the above-mentioned wound dressing was used. Then, as in group 1, gauze pads were placed on the ulcer, the limb was bandaged with a non-elastic supportive bandage and the compression short-stretch bandages were used. The Suprasorb^®^ A + Ag dressing was changed every day until the ulcer was healed. As in group 1, 25–35 mm Hg compression pressure was precisely applied with the help of the Kikuhime device pressure sensor.

At the baseline, and after 3 and 5 weeks of treatment, the wounds (from both groups) were swabbed for microbiological assessment. The tested material was inoculated onto a medium for selective bacterial culture and incubated for 24–48 h at 37 °C.

The culture for aerobic bacteria was performed with the following media: Columbia agar with 5% sheep blood, Chapman medium, selective-differentiating MacConkey medium (Becton, Dickinson and Company, Franklin Lakes, USA), and D-coccosel medium. Incubation was performed under aerobic conditions at a temperature between 35 and 37 °C and the bacteria growth was assessed after 48 h. Anaerobic bacteria were incubated for 4 to 5 days in an anaerobic atmosphere using Genoboxanaer (Bio Merieux, Warsaw, Poland) at 35–37 °C. In order to culture these bacteria Schaedlerbase (Neo) and Vanco substrate (Bio Merieux, Warsaw, Poland) were used.

The identification of isolated microorganisms was performed using standard microbiological methods, and the following were assessed: morphology using Gram-stained preparation, growth on selective media, morphology of colonies, and their biochemical and serological properties. To perform this, an ATB system with reagent kits (Bio Merieux) was used.

Prior to the treatment and during wound dressing and compression changes, the area of ulcers was measured. It was performed once a week until the wound healed completely. The procedure was carried out as follows: firstly, homothetic congruent projections of the ulcers were plotted onto transparent foil, after which planimetric measurements of the wounds were taken with the use of digitizer Mutoh Kurta XGT-1218A3 (Mutoh, Phoenix, AZ, USA) [56,57,58]. The speed with which the ulceration area was decreasing daily was calculated for each patient according to the formula:

v_s_ = (S_i−1_ − S_i_)/t

s (subscript): group number (1 or 2);
S_i_: ulceration area at the time of the previous measurement (one week earlier);
S_i−1_: ulceration area on the day of a given measurement;
t: time (in days) in which the ulceration area changed from S_i−1_ to S_i_ (7 days).

### 4.3. Statistical Analysis

Statistical characteristics of the continuous variables have been presented as arithmetic means and their standard deviations (SD) or as medians and interquartile ranges. The results were analyzed using the Mann–Whitney U-test; *p*-values < 0.05 were considered statistically significant. All calculations were carried out using Statistica 8 (software version 8, StatSoft, Cracow, Poland).

## 5. Conclusions

1. The application of Manuka honey in the treatment of non-healing, chronic VLUs is beneficial, as it significantly shortens the healing time of such wounds.
2. Manuka honey dressings reduce the surface of VLUs and, thus, shorten the time of treatment.
3. The use of Manuka honey dressings results in the faster removal of bacterial microbiota from VLUs

## Figures and Tables

**Table 1:** The number of new patients with completely healed ulcers depending on the duration of treatment (group 1—Manuka honey, group 2—standard treatment).

Group	Duration of Treatment (Weeks)													
	1	2	3	4	5	6	7	8	9	10	11	12	13	14
1		4	10	8	7	7	4							
2				6		6		7		7	5	4	3	2

**Table 2:** The incidence of chosen 7 bacteria species in tested ulcerations, measured as a number of patients with particular species being isolated from wounds.

Isolated Bacteria Species	Before Treatment		After 3 Week Treatment		After 5 Week Treatment	
	Group 1	Group 2	Group 1	Group 2	Group 1	Group 2
*Staphylococcus aureus*	35	36	18	29	0	15
*Escherichia coli*	33	32	11	21	0	12
*Pseudomonas aeruginosa*	25	26	10	17	0	11
*Proteus mirabilis*	23	25	11	15	0	12
*Streptococcus β-haemoliticus*	23	26	13	18	0	11
*Enterococcus faecalis*	22	22	10	17	0	9
*Klebsiella pneumoniae*	21	22	12	14	0	8

**Table 3:** Characteristics of the research groups; (group 1—Manuka honey; group 2—standard treatment).

	Group 1	Group 2
Number of patients	40	40
Women	27	26
Men	13	12
Age (years)	49–79	51–75
Ulcer location—left crus	14	16
Ulcer location—right crus	26	24
Ulcer area at the beginning (cm^2^)	6.8–17.4	6.6–17.8
Duration of ulceration (months)	17–29	18–31
Full mobility	30	29
Limited mobility	10	11
Ankle-brachial index (ABI)	0.92–1.11	0.96–1.12
Body mass index (BMI) (kg/m^2^)	23.6–39.7	24.8–40.2

**Table 4:** Classification of venous dysfunction (according to the CEAP classification); (group 1—Manuka honey, group 2—standard treatment).

CEAP Class	Group 1	Group 2
C_6_E_P_A_S2,3_P_R_	19	17
C_6_E_P_A_S3D18_P_R_	11	13
C_6_E_P_A_S3D18P18_P_R_	5	6
C_6_E_P_A_S4_P_R_	5	4

## Data Availability

The original contributions presented in this study are included in the article. Further inquiries can be directed to the corresponding author.
