# Correction: Kucharzewski et al. Topical Application of Manuka Honey for the Treatment of Non-Healing Venous Leg Ulcers. *Pharmaceuticals* 2025, *18*, 149

**DOI:** 10.3390/ph18111740

**Published:** 2025-11-17

**Authors:** Marek Kucharzewski, Kinga Spyrka, Ewa Rojczyk, Jakub Brela

**Affiliations:** 1Wladyslaw Bieganski Collegium Medicum, Jan Dlugosz University in Czestochowa, 42-200 Czestochowa, Poland; kucharzewskimarek@poczta.onet.pl (M.K.); ikingaxd@gmail.com (K.S.); j.brela@ujd.edu.pl (J.B.); 2Surgical Outpatient Clinic, John Paul II District Hospital in Włoszczowa, 29-100 Włoszczowa, Poland; 3Faculty of Medicine, Academy of Silesia in Katowice, 40-555 Katowice, Poland

## References

In the original publication [1], the reference 37 was retracted. The authors decided to deleted the reference 37 and related sentence in Section “3. Discussion”, as well as the reference 38 in the sentence. The deleted sentence is showed below:

“A hydrocellular foam dressing impregnated with either Manuka honey (n = 54) or an amorphous hydrogel (n = 54) on venous ulcers of longer duration, covered with at least 50% slough, was used by Gethin and Cowman. It was observed that those patients had larger venous ulcers than the patients studied by Jull et al., despite a weekly application of dressings with up to four layers of compression bandaging for a time span of 4 weeks [37]. After using Margolis’ [38] standardized adjustment for venous ulcer areas > 5 cm^2^ and a duration > 6 months, a greater percentage of patients in the Manuka honey group (*p* = 0.025) recovered at the 12-week period (*p* = 0.037) compared to the hydrogel control group, and earlier epithelialization (*p* = 0.042) was observed [37].”

With this correction, the order of some references has been adjusted accordingly. This correction was approved by the Academic Editor, and the authors state that their scientific conclusions remain unaffected. The original publication has also been updated.
